# Hospital Laboratory Survey for Identification of *Candida auris* in Belgium

**DOI:** 10.3390/jof5030084

**Published:** 2019-09-05

**Authors:** Klaas Dewaele, Katrien Lagrou, Johan Frans, Marie-Pierre Hayette, Kris Vernelen

**Affiliations:** 1 Department of Clinical Microbiology, Imelda General Hospital, 2820 Bonheiden, Belgium; 2 Department of Laboratory Medicine and National Reference Centre for Mycosis, University Hospitals of Leuven, 3000 Leuven, Belgium; 3 Department of Microbiology, Immunology and Transplantation, KU Leuven, 3000 Leuven, Belgium; 4 Department of Clinical Microbiology and National Reference Center for Mycosis, University Hospital of Liège, 4000 Liège, Belgium; 5 Quality of Laboratories, Sciensano, 1050 Brussels, Belgium

**Keywords:** *Candida auris*, *Candida haemulonii*, laboratory survey, MALDI-TOF, Vitek YST ID

## Abstract

*Candida auris* is a difficult-to-identify, emerging yeast and a cause of sustained nosocomial outbreaks. Presently, not much data exist on laboratory preparedness in Europe. To assess the ability of laboratories in Belgium and Luxembourg to detect this species, a blinded *C. auris* strain was included in the regular proficiency testing rounds organized by the Belgian public health institute, Sciensano. Laboratories were asked to identify and report the isolate as they would in routine clinical practice, as if grown from a blood culture. Of 142 respondents, 82 (57.7%) obtained a correct identification of *C. auris*. Of 142 respondents, 27 (19.0%) identified the strain as *Candida haemulonii*. The remaining labs that did not obtain a correct identification (33/142, 23.2%), reported other yeast species (4/33) or failed to obtain a species identification (29/33). To assess awareness about the infection-control implications of the identification, participants were requested to indicate whether referral of this isolate to a reference laboratory was desirable in a clinical context. Over one-third of all respondents (54/142, 38.0%) stated that they would not send the isolate to a reference laboratory, neither for epidemiological reasons nor for identification confirmation or antifungal susceptibility testing. This comprised 41.5% of the laboratories that submitted an identification of *C. auris* (34/82). Awareness among Belgian microbiologists, therefore, remains inadequate more than two years after *C. auris*’ emergence in European clinics. Our data confirm high rates of misidentifications with commonly used identification methods. Programs for raising awareness in European hospitals may be warranted.

## 1. Introduction

The emergence of unusual, opportunistic yeast species has been significant in recent years [1]. *Candida auris* is unique in the scale of its expansion and in the persistency of the nosocomial outbreaks it has readily caused. Several commonly used identification systems were enabled for identification of this species only in 2018. Unsurprisingly, in preceding years this yeast has often been misidentified [2]. In addition, unaware microbiologists may disregard this *Candida* species’ unusual ability for spreading and persisting in the nosocomial environment. Sporadic identifications may thus be discarded and/or inadequately reported to clinicians and public health authorities. With the first reported cases in Europe and the U.S. in 2016, surveillance bodies called for vigilance with regard to this unfamiliar pathogen [3,4]. However, as of 2019, not much data exist on laboratory preparedness, in terms of both identification capacity and awareness about infection-control implications of the identification [5]. To assess preparedness of Belgian clinical laboratories, we conducted a laboratory survey with a blinded *C. auris* strain. In addition to the laboratories’ identification capacity, we inquired for awareness about this yeast’s nosocomial risk profile by requesting participants to assess the need for referral of this strain to a reference laboratory. This paper summarizes the findings of this survey.

## 2. Materials and Methods

Sciensano is the Belgian national public health institute. Its activities include quality assessment of clinical laboratories. Annually, four microbiology quality assessment rounds are organized. Participants include all licensed clinical laboratories in Belgium and Luxembourg, comprising 137 and 8 laboratories, respectively. Participants are provided with blinded lyophilized samples and with a clinical context to guide elaboration of the sample and interpretation of the identification obtained. In April 2018, a *C. auris* strain was included in a panel of four strains. The isolate originated from a sporadic case of *C. auris* sepsis in a patient that was transferred from a Kuwaiti to a Belgian hospital in 2016 [6]. At the first identification attempts, inconsistent identifications were obtained using different commonly used commercial systems. In the survey, participants were instructed to identify and report the strain as if grown from a blood culture in a patient transferred from Kuwait. Additionally, as for all strains in the panel, participants were asked to assess the need for referral of the isolate to a reference laboratory and to add information about the reason why they would do so, i.e., confirmation of the identification and/or antifungal susceptibility testing results, and/or for epidemiological reasons. Responses were submitted via an online form. No identification confidence scores or antifungal susceptibility testing data were collected. Participants received the survey results and an educational commentary in a yearly report released in October 2018. Three of 145 participating laboratories (2.1%) do not perform in-house fungal identifications and did not submit an identification. 

## 3. Results

**Figure 1:**
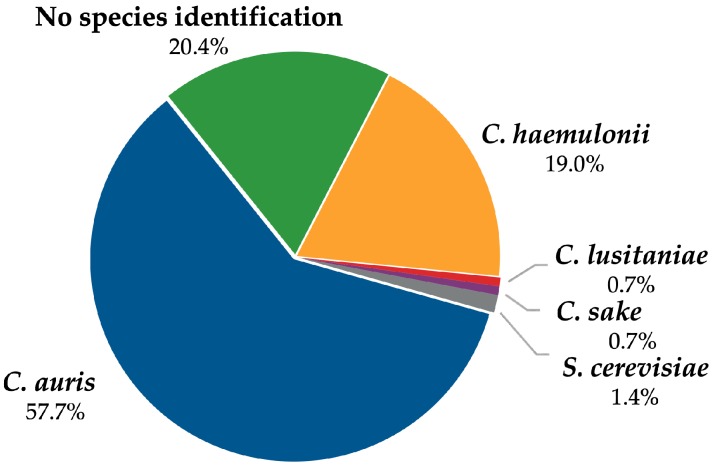
Identifications provided by 142 labs.

Of 142 respondents, 19.0% (27) identified the blinded strain as *Candida haemulonii* (Figure 1). 20.4% (29) did not obtain a species-level identification (that is, reported ‘yeast’, ‘*Candida* species’, or ‘*Candida* non-*albicans*’). Other species misidentifications accounted for less than 3% of survey responses (*Candida lusitaniae* 0.7%, *Candida sake* 0.7%, *Saccharomyces cerevisiae* 1.4%).

Of 142 participants, 57.7% (82 laboratories) obtained a correct identification of *Candida auris*. Most of these (71/82) used a MALDI Biotyper system (Bruker Daltonics, Bremen, Germany) (Table 1). One laboratory that obtained *C. auris* used an unspecified MALDI-TOF MS system. Other techniques that yielded a correct identification were the biochemical YST ID cards for Vitek 2 with library version 8.01 (bioMérieux, Marcy-l’Étoile, France) (9/82 participants) or 18S rRNA sequencing (one participant). 

Misidentifications as *Candida haemulonii* (27/142 participants, 19.0%) were obtained with the Vitek 2 YST ID system with library version 7.01 (23/27 laboratories) and the Vitek MS system (bioMérieux) (4/27 participants). Participants that submitted a genus-level identification or ‘yeast’ (29/142 respondents, 20.4%) used API methods or custom biochemical methods (15/29 laboratories), the Vitek MS system (9/29 laboratories), the Vitek YST ID system (4/29 participants), or a MALDI Biotyper system (one participant). Other species misidentifications (4/142 respondents, 2.8%) were obtained by traditional biochemical and/or phenotypic methods (*Candida sake* and twice *Saccharomyces cerevisiae*) or Vitek MS (*Candida lusitaniae* by one respondent).

In addition to the strain’s identification, all participants were requested to indicate whether referral of this isolate to a reference laboratory was desirable in a clinical context (i.e., isolate cultured from blood). Possible reasons that could be marked were epidemiological reasons, confirmation of the identification, and/or confirmation of antifungal susceptibility testing results. Over one-third of all participants, regardless of the identification obtained, would not send the isolate to a reference center for any reason (54/142, 38.0%); this was about half of the respondents of *C. haemulonii* (13/27), 17.2% of laboratories that did not obtain a species-level identification (5/29), and two of four laboratories that obtained another identification than *C. auris* or *C. haemulonii*. Of 82 labs identifying *C. auris*, 41.5% (34) would not submit the strain to a reference laboratory for any reason (Table 2).

## 4. Discussion

Conducted in April 2018, this laboratory survey demonstrated a significant rate of misidentifications and failed identifications of *C. auris* in laboratories in Belgium and Luxembourg. Of 142 laboratories, 42.3% (60) did not obtain a species-level identification, or reported a misidentification. These laboratories employed various commonly used identification systems. Of 142 respondents, 57.7% (82) submitted a correct identification of *C. auris*. This mainly comprised users of a MALDI-TOF MS system (72/82). A smaller number of laboratories obtained the identification using the Vitek 2 YST ID system (library version V. 8.01) (9/82 labs) or with 18S rRNA sequencing (1/82 labs).

Among MALDI-TOF MS users, divergent results were obtained with bioMérieux’s Vitek MS system and with the Bruker MALDI Biotyper. While nearly all MALDI Biotyper users submitted a correct identification (71/72 Bruker users), the Vitek MS system yielded no species-level identification (9/14 Vitek MS users), or a misidentification of *Candida haemulonii* or *Candida lusitaniae* (4/14 and 1/14 Vitek MS users, respectively). These findings concur with results of an earlier MALDI-TOF MS survey of 125 international laboratories [7]. Sherry et al. reported an overall identification rate of 69.8%, with a significantly higher proportion of Vitek MS users reporting failed identifications or misidentifications of *C. auris* [7]. The poorer performance of the Vitek MS system in the present survey is explained by the absence of *C. auris* reference spectra in its routinely used (CE-IVD-approved) reference library (Knowledge Base version 3.0) (Table 3). The first CE-IVD-approved library that contains *C. auris* spectra (Knowledge Base V. 3.2) was released in the summer of 2018, which was after the survey. *Candida auris* spectra were present in the ‘research-use only’ (RUO) SARAMIS library (V. 4.14) with the ‘Saccharomycetaceae update’, released in 2016. This library, however, must be acquired and operated separately from the CE-IVD library. No clinical laboratories in Belgium used it at the time of the survey. 

For Bruker’s MALDI Biotyper CA platform, used in the United States, the first FDA-approved library allowing *C. auris* identification was released in April 2018 (FDA and RUO library version 8.0). However, for the MALDI Biotyper IVD system, CE-IVD-approved libraries containing *C. auris* spectra were in use in Europe since December 2013 (library version 4.0 and onward) (Table 4). All surveyed laboratories that used a MALDI Biotyper system, therefore, disposed of a library containing *C. auris* reference spectra. Although identification confidence scores were not asked for in the survey, many Bruker users nevertheless reported difficulties in reaching acceptable species-level identification scores (that is, scores ≥2.00, according to Bruker recommendations). A full-tube extraction procedure did not significantly improve identification scores. Some Biotyper users obtained the identification through support of the manufacturer (employing at that time noncommercially available libraries). The suboptimal performance of the Biotyper in this survey is likely related to the limited number of *C. auris* reference spectra in its libraries that were from strains of different geographic origins than the survey strain. Until the release of library version 8.0 in 2018, Bruker libraries contained spectra from only three *C. auris* strains of East-Asian origin. The survey strain was originally isolated in a patient transferred from a Kuwaiti hospital, and thus likely belongs to a Middle Eastern clade. Different geographic clades display spectral differences, which we suspect may result in suboptimal confidence scores [8]. Several additional variables may also affect performance of MALDI-TOF MS for yeast identifications [9]. Independent of the survey, the quality control strain was relatively easily identified with the updated MALDI Biotyper version 8.0 library (confidence scores > 2.00 with on-plate extraction with formic acid). This library contains six added spectra from geographically diverse *C. auris* strains. Employing the CDC’s online MicrobeNet library (freely available to Bruker users), high-confidence scores (>2.00) were obtained without the need of any (partial or full-tube) extraction procedure. The online ‘MSI platform’ for fungal identification allowed high-confidence identification of our strain using the MALDI Biotyper, after full-tube extraction [10]. The release of adapted libraries, therefore, holds promise for reliable identification of *C. auris* by MALDI-TOF MS users [11,12,13]. Nonetheless, the example of *C. auris*’ emergence demonstrates that the use of the more comprehensive ‘research-use only’ libraries in clinical practice may be required for obtaining a rapid (presumptive) identification of emerging pathogens.

Commonly used biochemical systems have often yielded failed identifications or misidentifications of *C. auris* [2]. Misidentifications often involve species of the *Candida haemulonii* complex, of which *C. auris* is counted a member [2,18]. In the present survey, *C. haemulonii* was obtained mainly by the Vitek YST ID system with library version V. 7.01 (23/27 laboratories), and by a smaller number of participants, using the Vitek MS system (4/27 laboratories). Shortly prior to the survey, the updated Vitek YST ID library version V. 8.01 had been released. This was the first library version of the Vitek YST ID system capable of identifying *C. auris*. Laboratories that had already implemented this update obtained a correct identification of the survey strain. Nevertheless, some authors have reported continued misidentifications of certain *C. auris* strains with this library version [19,20]. Identifications of *Candida haemulonii*, *Candida duobushaemulonii*, or *Candida* species require confirmatory testing. 

Traditional biochemical systems were used by a minority of participants in the survey (18 of 142 participants); these concerned API 20C AUX (bioMérieux), AuxaColor strips (Bio-Rad Laboratories, Hercules, CA, USA), or custom biochemical methods. These mostly failed to deliver a species-level identification (15/18 laboratories). As previously reported, some yielded misidentifications of *Candida sake* (1/18) or *Saccharomyces cerevisiae* (2/18) [2]. 

In May 2019, independent of the identification survey, the same 145 laboratories were polled for the prevalence of identifications of *C. haemulonii* and *C. duobushaemulonii* in the preceding years, since these may have represented misidentifications of sporadic *C. auris* cases. Of 62 responding laboratories, almost one-third (19/62) identified one or both species during a variable timespan in the past. Several of these identifications were obtained with a Bruker MALDI Biotyper system with a recent library (that is, V. 5.0 or later), which can be considered reliable to distinguish these species from *C. auris* [21]. Therefore, species of the *C. haemulonii* complex appear to be prevalent in our area and care must be taken to differentiate the members of this group accurately. Further retrospective studies of stocked yeast isolates and reanalysis of raw identification data of relevant species may be useful for epidemiological purposes. 

As of 2019, the large majority of hospitals in Belgium and Luxembourg have access to relatively reliable identification systems, if recent system updates are implemented. Over 60% of participants (87/145) dispose of a MALDI-TOF MS system (bioMérieux or Bruker), and an additional 24.8% (36/145) use the Vitek YST ID system. However, we observed inadequate familiarity of Belgian microbiologists with the epidemiological and infection-control implications of obtaining an identification of *C. auris*. When asked whether referral to a reference laboratory would be indicated for any reason, over one-third of all respondents (54/142 laboratories, 38.0%) answered negatively. Of the group that submitted the identification of *C. auris*, this represented 41.5% (34/82). Of the group that did not obtain a species-level identification, this represented 17.2% (5/29). This reveals that a significant proportion of Belgian laboratory professionals remain uninformed about the importance of accurate identification and reporting (to clinicians and public health authorities) of this pathogen in particular, and some of *Candida* bloodstream isolates in general. Reporting *Candida* bloodstream isolates at the genus level only and without susceptibility testing in clinical practice is unacceptable. Belgian diagnostic laboratories can be run by clinical biologists with very limited microbiology training (the legal minimum of a microbiology internship is six months), which can be an explanatory factor. In addition, mycology is often underrepresented in the microbiology training. 

International data on awareness of *C. auris’* risk profile among laboratory professionals are scarce. Sherry et al., in the aforementioned international *C. auris* survey, noted that 24% of laboratories that had obtained the correct identification of *C. auris* rather reported *Candida* spp. [7].

In response to the large-scale hospital outbreaks occurring in the U.K. and Spain in 2016, various initiatives have been taken to improve awareness in Europe. The European Centers for Disease Control (ECDC) published a ‘rapid risk assessment’ on *C. auris* in December 2016 [3]. Kohlenberg et al. queried national availability of a mycology reference center and public health measures taken in European countries from 2013 to 2017 [5]. Many countries, Belgium included, lacked a laboratory and/or clinical alert to relevant healthcare personnel about *C. auris*’ emergence. In Belgium, healthcare institutions were only indirectly notified through a mention in an infectious diseases bulletin in January 2017. To monitor awareness and guide further initiatives, a follow-up survey with a blinded *C. auris* strain is to be conducted in 2019. Our data suggest that further national initiatives may be required for attaining adequate awareness about *C. auris* in European clinical laboratories.

## 5. Conclusions

*Candida auris* has rapidly become a pathogen of global public health concern. Clinically approved libraries for several commonly used identification systems have been released only recently. Conducted in April 2018, two years after the first (and only) detected Belgian *C. auris* case, our survey demonstrates significant rates of misidentifications in laboratories in Belgium and Luxembourg. We also find that awareness about the nosocomial risk profile of *C. auris* is inadequate. Nevertheless, a large majority of laboratories in Belgium and Luxembourg have the ability to identify *C. auris* when recently released identification system updates are implemented. Further initiatives for improving awareness may be warranted.

## Figures and Tables

**Table 1:** Number of respondents of a given submitted identification employing a given identification method.

	*C. auris* (*n* = 82)	*C. haemulonii* (*n* = 27)	No Species Identification (*n* = 29)	Other Species Identification (*n* = 4)
Bruker MALDI Biotyper ^1^	71 (86.6%)	-	1 (3.4%)	-
bioMérieux Vitek YST ID ^2^	9 (11.0%)	23 (85.2%)	4 (13.8%)	-
bioMérieux Vitek MS ^3^	-	4 (14.8%)	9 (31.0%)	1 (25.0%)
Traditional biochemical methods	-	-	15 (51.7%)	3 (75.0%)
Other	2 (2.4%)	-	-	-

^1^ Employing a research-use only (RUO) or an in vitro diagnostics CE-approved library (CE-IVD). ^2^ Using Vitek YST ID library version V. 7.01 or V. 8.01. ^3^ Using a CE-IVD-approved library. Hyphen (-) indicates that no respondents used the method concerned for a given identification.

**Table 2:** Proportion of respondents per identification that would refer the isolate to a reference laboratory and justifications for the decision.

Submitted Identification	Referral of Strain?		Reason(s) for Referral		
	Yes	No	ID and/or AFST Confirmation	Epidemiological Reasons	ID and/or AFST Confirmation and Epidemiological Reasons
*C. auris* *n* = 82	48/82 (58.5%)	34/82 (41.5%)	23/48 (47.9%)	8/48 (16.7%)	17/48 (35.4%)
No species ID *n* = 29	24/29 (82.8%)	5/29 (17.2%)	22/24 (91.7%)	-	2/24 (8.3%)
*C. haemulonii* *n* = 27	14/27 (51.9%)	13/27 (48.1%)	13/14 (92.9%)	-	1/14 (7.1%)
Other species ID *n* = 4	2/4 (50.0%)	2/4 (50.0%)	2/2 (100.0%)	-	-

Abbreviations: ID, identification; AFST, antifungal susceptibility testing. Hyphen (-) indicates no respondents submitted this reason for referral of the isolate to a reference laboratory.

**Table 3:** Library versions of the bioMérieux Vitek MS system and number of *C. auris* reference strains included [14,15].

Release Date ^1^	Library and Version	RUO/CE-IVD	No. of *C. auris* Reference Strains Included
April 2015	SARAMIS V. 4.13	RUO	None
June 2016	Knowledge Base V. 3.0	CE-IVD	None
June 2016	SARAMIS V. 4.14	RUO	None
July 2016	SARAMIS V. 4.14 Saccharomycetaceae update	RUO	20
December 2017	SARAMIS V. 4.15	RUO	20
August 2018	Knowledge Base V. 3.2	CE-IVD	20

^1^ Release dates may vary per region. Abbreviations: RUO, research-use only; CE-IVD, CE-approved for in vitro diagnostic use.

**Table 4:** Library versions of the Bruker MALDI Biotyper IVD system and number of *C. auris* reference strains included [16,17].

Release Date ^1^	Library and Version	RUO/CE-IVD	No. of *C. auris* Reference Strains Included
December 2013	MBT Compass/IVD Library DB-5627 (V. 4)	RUO/CE-IVD ^2^	3
June 2015	MBT Compass/IVD Library DB-5989 (V. 5)	RUO/CE-IVD ^2^	3
April 2016	MBT Compass Library DB-6903 (V. 6)	RUO	3
	MBT IVD Library DB-6763 (V. 6)	CE-IVD	3
February 2017	MBT Compass Library MBT-7311 (V. 7)	RUO	3
	MBT IVD Library DB-7171 (V. 7)	CE-IVD	3
April 2018	MBT Compass Library MBT-7854 (V. 8)	RUO	9
	MBT IVD Library DB-7712 (V. 8)	CE-IVD	9
April 2019	MBT Compass Library MBT-8468 (V. 9)	RUO	9
	MBT IVD Library DB-8326 (V. 9)	CE-IVD	9

^1^ Release dates may vary per region. ^2^ RUO and CE-IVD-approved libraries were identical in version V. 4 and V. 5. Abbreviations: RUO, research-use only; CE-IVD, CE-approved for in vitro diagnostic use.
